# In-Field Wheat Reflectance: How to Reach the Organ Scale?

**DOI:** 10.3390/s22093342

**Published:** 2022-04-27

**Authors:** Sébastien Dandrifosse, Alexis Carlier, Benjamin Dumont, Benoît Mercatoris

**Affiliations:** 1 Biosystems Dynamics and Exchanges, TERRA Teaching and Research Center, Gembloux Agro-Bio Tech, University of Liège, 5030 Gembloux, Belgium; alexis.carlier@uliege.be (A.C.); benoit.mercatoris@uliege.be (B.M.); 2 Plant Sciences, TERRA Teaching and Research Center, Gembloux Agro-Bio Tech, University of Liège, 5030 Gembloux, Belgium; benjamin.dumont@uliege.be

**Keywords:** leaves, ears, phenotyping, multispectral, camera, image, spectrometer

## Abstract

The reflectance of wheat crops provides information on their architecture or physiology. However, the methods currently used for close-range reflectance computation do not allow for the separation of the wheat canopy organs: the leaves and the ears. This study details a method to achieve high-throughput measurements of wheat reflectance at the organ scale. A nadir multispectral camera array and an incident light spectrometer were used to compute bi-directional reflectance factor (BRF) maps. Image thresholding and deep learning ear detection allowed for the segmentation of the ears and the leaves in the maps. The results showed that the BRF measured on reference targets was constant throughout the day but varied with the acquisition date. The wheat organ BRF was constant throughout the day in very cloudy conditions and with high sun altitudes but showed gradual variations in the morning under sunny or partially cloudy sky. As a consequence, measurements should be performed close to solar noon and the reference panel should be captured at the beginning and end of each field trip to correct the BRF. The method, with such precautions, was tested all throughout the wheat growing season on two varieties and various canopy architectures generated by a fertilization gradient. The method yielded consistent reflectance dynamics in all scenarios.

## 1. Introduction

The term “reflectance” refers to the proportion of light that is reflected by an object. It generally varies with the wavelength of the incident light. It is an absolute quantity that is comparable between different irradiance levels and sensors. In field phenotyping, spectral reflectance is used to derive the architecture or physiology of the crops. The sensors can be carried by unmanned aerial vehicles (UAVs), ground vehicles, and platforms or human operators [1]. Satellites and aircrafts are primarily used in precision agriculture, where it is not necessary to distinguish experimental micro-plots, but they could be useful in large-scale phenotyping approaches [2]. Extracting information at the plant-organ scale requires close-range vectors such as ground-based approaches or low altitude UAVs. Nevertheless, in practice, reflectance at the organ scale has almost exclusively been studied in controlled conditions, where the reflectance of single isolated organs could be directly related to their anatomy and physiology; it has, for example, been exploited to detect nitrogen content [3] and diseases [4,5,6]. In the field, reflectance measured at the canopy scale depends not only on organ anatomy and physiology but also on sun-sensor view geometry, canopy architecture, and soil background [7,8,9,10,11,12,13]. To further complicate the problem, all those factors have different impacts depending on the development stage of the crop [14]. One approach to extract crop properties based on canopy reflectance is to exploit vegetation indices [15], i.e., mathematical operations combining reflectance from several wavebands. These indices are sometimes complex and supposed to be sensitive to crop variables of interest while being relatively robust to the confounding factors. To extract useful agronomic information from reflectance measured at the canopy scale, another approach is to exploit models. Many types of models can be built [16], and one of the most widely-used is PROSAIL [17,18,19] (PROSPECT [20] + SAIL [21]). This model integrates characteristics of the incident light and canopy architecture to link canopy reflectance to leaf biochemical content. Several versions of the two components of the model have been developed, and many researchers have discussed them [18,22]. Wheat canopies are composed of several types of organs: stems, leaves, and ears; however, PROSAIL works on leaves, but ears contain unwanted information. The authors of [23] showed that canopies with and without ears presented different reflectance properties, and the authors of [24] observed that the proportion of ears in the nadir-viewed canopy elements correlated with deviations between PROSAIL spectral outputs and field spectral measurements. These studies highlight a broader issue: even at close-range, the current in-field approaches provide reflectance values for entire crop canopies without distinctions between plants and soil and between plant organs. To understand the reason for this issue, it is necessary to examine the reflectance measurement methods that have been used for crop phenotyping. 

To compute a reflectance value, one needs to possess information on not only the light reflected from the plants but also the incident light. Active sensors solve this issue by including a light emitter. Therefore, the incident light possesses known characteristics. The reflectance sensors of this category are non-imaging devices. There are many commercial models such as the GreenSeeker (Trimble Navigation Ltd., Sunnyvale, CA, USA) [25,26,27,28,29,30], the N-Sensor ALS (YARA international ASA, Oslo, Norway) [25,27,29], the CropCircle (Holland Scientific Inc., Lincoln, NE, USA) [26,27,29,30], and the CM 1000 (SDEC, Reignac-sur-Indre, France). Those farmer-oriented sensors rely on reflectance measurements but only provide vegetation index values. Moreover, their wavebands are limited by the light source. They can be used on a specific organ to measure its reflectance, but they require a human or a machine to position them.

Passive reflectance sensors, on the other hand, work thanks to the incident sunlight. Whether these sensors are imagers or not, two main methods are used to account for the variations of incident light. The first method relies on a reference panel of known reflectance spectrum and properties. It was used along with non-imaging sensors in [31,32,33]. The panel can be imaged before and after each canopy spectrum acquisition, but this does not account for changing sunlight conditions between those reference measurements. If the sensor is an imager, it is also possible to include the panel in the observed canopy [34]. This has the advantage of being able to collect the reference reflectance at the exact time of the canopy image capture. However, it also reduces the size of the studied plant zone and limits the throughput of the system. Most studies exploiting a camera to measure reflectance used such a panel of known reflectance [23,34,35,36,37,38,39,40]. The second method to account for the incident light deploys a second sensor facing the sky in addition to the sensor facing the canopy. At close range, it is mainly used for spectrometers. The up-facing probe can be exploited to compute reflectance [28,41,42] or to compensate for variations in sunlight if the reflectance is derived from a reference panel acquired before or after the canopy spectra [43]. That principle of up- and down-facing probes has been integrated in a diversity of commercial devices such as the HandySpec Field (tec5, Oberusel, Germany) used by [43,44,45]. Concerning imaging devices, several commercial solutions have coupled an incident light sensor (ILS) and a multispectral camera array: the ILS of the MAIA camera (SAL Engineering, Russi, Italy), the smart ILS of the MCA and ADC cameras (Tetracam Inc., Gainesville, FL, USA) [46], the SEQUOIA sunshine sensor (Parrot SA, Paris, France), and the MicaSense DLS 2 (MicaSense Inc., Seattle, WA, USA). Reflectance computations are possible with associated proprietary software or custom scripts. The Micasense company uploaded detailed Python code on Github (https://github.com/micasense/imageprocessing, accessed on 25 April 2022). A documented UAV approach was proposed by [47] for forest reflectance measurements. 

It appears that most of the wheat reflectance measurements have been carried out by non-imaging devices such as spectrometers or by high altitude cameras with limited spatial resolution. Reflectance measurements performed with close-range imagers rely on bulky reference panels, and the image processing step often does not include the separation of organs. A method to isolate the reflectance of shaded and sunlit wheat leaves was used by [48], but their homemade reference panel, of unknown reflectance, only permitted relative reflectance measurements. In addition, the positioning of the panel in the images turned out to be complicated, especially because of the shadows caused by their acquisition platform. The authors of [49] exploited a hyperspectral camera to extract the reflectance of wheat leaves and ears, shaded or sunlit, but the imaging of the reference panel added a constraint, and extraction was only performed every 15 min despite natural lighting conditions changing much faster. For tobacco and sugar beet, Refs. [34,50], respectively, managed to obtain the reflectance of leaves separately from the soil using a hyperspectral camera and a reference panel. 

This paper details a clear and simple method based on a multispectral camera array and an incident light sensor to compute the reflectance of wheat organs separately and without the need for a reference panel in each image. The reference panel is only used once before the measurement campaign to build the response curves of the cameras, and once at the beginning and once at the end of each field trip. During a field trip, the incident light sensor and the camera response curves allow for the computation of the reflectance. To validate the method, the evolution of the reflectance measured on a reference panel and wheat organs was studied on same wheat area throughout the day at six wheat growing stages. Finally, the approach was tested in two fertilization trials all over the growing season to assess its consistency for the two different varieties and the contrasted architectures generated by the fertilization gradient. 

## 2. Materials and Methods

### 2.1. Material

The multispectral camera array was a Micro-MCA (Tetracam Inc., Gainesville, FL, USA) consisting of six monochrome cameras equipped with 1280 × 1024 pixel CMOS sensors. The cameras were mounted with narrow band-pass optical filters centered at 490, 550, 680, 720, 800, and 900 nm. Each band had a width of 10 nm except for the 900 nm band, which had a width of 20 nm. The lenses had a focal length of 9.6 mm and an aperture of f/3.2. Additionally, a pair of GO-5000C-USB RGB cameras (JAI A/S, Copenhagen, Denmark) was used to record color and 3D information by stereovision. The distance between the centers of the two sensors was 50 mm. Each device was equipped with a 2560 × 2048 pixel CMOS sensor and an LM16HC objective (Kowa GmbH, Düsseldorf, Germany). Their focal length was 16 mm, and the aperture was set to f/4.0. The ILS was an AvaSpec-ULS2048 equipped with a cosine corrector (Avantes, Apeldoorn, The Netherlands). Its signal-to-noise ratio was 200:1. The irradiance calibration was carried out in the factory on 23 March 2020. Finally, the reference panel was an MAPIR camera reflectance calibration target V2 (MAPIR Inc., San Diego, CA, USA). The panel consisted of four targets of a felt-like material: black, dark gray, light gray, and white targets. A reflectance spectrum was provided for each target. According to the manufacturer, the targets presented no anisotropy because of the diffuse properties of the material.

### 2.2. Data Acquisition

#### 2.2.1. Multi-Sensor System Set-Up

The multi-sensor platform was designed to capture nadir frames of wheat in field conditions. The sensor pod was positioned on a cantilever beam to avoid shadows from the rest of the platform in the images. The height of the pod was adjusted at each field trip to keep a distance around 1.6 m between the cameras and the top of the wheat plants. At this distance, the image footprint was 0.98 m² for the multispectral device cameras and 1.26 m² for the RGB cameras. The images were originally recorded using a color depth of 10 or 12 bits per pixel but later reduced to 8 bits per pixel because the stereovision and registration open-source libraries require 8-bit inputs. The auto-exposure algorithms of RGB and multispectral devices were adapted to prevent image saturation (Appendix A). The ILS was placed at the top of the acquisition platform. A spectrum of the incident light was recorded at each image acquisition using the 16-bit resolution of the analog-to-digital converter. Each saved spectrum was the average of three consecutive measurements. It was corrected for the non-linearity of pixel response to exposure time and dark noise. Thanks to the factory calibration, digital values were converted to irradiance data. Each acquisition of images and their associated solar spectrum took a few seconds, corresponding to the time to average the spectra and compute a proper exposure time for all cameras. 

#### 2.2.2. All-Day-Long Acquisitions

The purpose of the acquisitions was to study the evolution of wheat reflectance throughout the day on a defined wheat zone. The experiment was repeated at several dates during the 2021 season. At all the concerned dates, the acquisition platform was positioned at the same place. It was located in Gembloux, Belgium (50°33′50″ N and 4°42′00″ E). The plot was planted with winter wheat (*Triticum aestivum* L., variety ‘KWS Dorset’) on 13th November 2020 with a density of 400 grains/m². The previous crop was winter wheat. Images and incident light spectra were acquired at six important BBCH crop development stages [51] and in a diversity of sky conditions (Table 1). The acquisitions were performed between 9 a.m. and 5 p.m. Every quarter of an hour, four acquisitions were performed at 10 s intervals: one in which the reference panel was placed above the canopy and three of the canopy without the reference panel. 

#### 2.2.3. Acquisitions in Fertilization Trials

Images and incident light spectra were acquired during the 2021 season in two field trials. Contrary to the all-day-long acquisitions (Section 2.2.2), the platform was moved over several micro-plots. The aim was to test the reflectance measurement method in a diversity of canopy development scenarios. The plots of the trials were located in Lonzée, Belgium (50°32′47″ N and 4°44′07″ E). The first trial (trial 1) was planted with winter wheat (*Triticum aestivum* L., variety ‘Mentor’) on 20 October 2020 with a density of 275 grains/m². The second trial (trial 2) was planted with winter wheat (variety ‘LG Vertikal’) on 27 October 2020 with a density of 300 grains/m². For both trials, the previous crop was potato. The experimental micro-plots measured 1.95 m × 6 m, and the row spacing was 0.14 m. The micro-plots were fertilized three times (at BBCH stages 28, 30, and 39) with 27% ammonium nitrate. Trial 1 consisted of a randomization of eight objects combining contrasted nitrogen inputs; eight replicates of each object were imaged. Trial 2 consisted of a randomization of sixteen objects combining contrasted nitrogen inputs and fungicide applications (0, 1, 2, or 3 dates of fungicide treatment); four replicates of the objects were imaged. Images of trial 1 were acquired on 10 March, 8 April, 15 April, 21 April, 28 April, 3 May, 11 May, 31 May, 9 June, 14 June, 25 June, 2 July, 8 July, 12 July, and 20 July. Images of trial 2 were acquired on 12 March, 25 March, 9 April, 15 April, 21 April, 28 April, 3 May, 11 May, 2 June, 11 June, 16 June, 25 June, 2 July, 9 July, 12 July, and 20 July. At each date and for each camera, four images were taken by micro-plot except for half of the trial 1 replicates that were dedicated to punctual destructive measurements, of which only two images were taken. In addition, two images of the reference panel were acquired, one at the beginning and one at the end of the data acquisition in a field trial.

### 2.3. Reflectance Computation

#### 2.3.1. Theoretical Basis

The reflectance of a plant surface involves a directional aspect, depending on the azimuth and zenith angles of the light source and the camera relative to the surface. The bi-directional reflectance factor (BRF) was defined by [52] as “the ratio of the radiant flux actually reflected by a sample surface to that which would be reflected into the same reflected-beam geometry by an ideal (lossless) perfectly diffuse (Lambertian) standard surface irradiated in exactly the same way as the sample”. It is a function of the wavelengths of the light source and the direction of the camera and the light source (azimuth and zenith angles). Mathematically, the BRF can be written as:

(1) $$\rho \left(\omega_{i},\omega_{r},\lambda \right)=\frac{L\left(\omega_{r},\;\lambda \right)\;\pi }{E\left(\omega_{i},\lambda \right)}$$

where L is the reflected radiance (W/(m^2^ sr)), E is the incident irradiance (W/m^2^), λ is the wavelength, ω_i_ is the direction of the light source, and ω_r_ is the direction of the camera. The BRF, as formulated here, is a physical quantity that cannot be measured, also known as a conceptual quantity. To be defined as “directional”, the formula considers infinitesimal solid angles, which cannot include measurable amounts of light flux. The measurable reflectance quantities necessitate a conical or hemispherical geometry [53]. In natural conditions, the incident sunlight is a combination of direct and diffuse irradiance, so it integrates the hemispherical aspect. From a strictly theoretical point of view, the quantity measured in this paper was actually an approximated BRF. In practice, as for previous studies in the plant domain, the term BRF is used.

#### 2.3.2. Camera Response Curves

The response curve of a camera is the relation between the digital numbers (DNs) in the images and the corresponding exposures (J/m^2^) at the sensor. That relation depends on camera electronics. It may not be perfectly linear. Considering that one exposure value is associated with each pixel, the term exposure map is used. The exposure map at the sensor is:

(2) $$H=E_{\mathrm{img}}\;t$$

where t is the exposure time (s) and E_img_ is the image irradiance map (W/m^2^):

(3) $$E_{\mathrm{img}}=\frac{L\;\pi \;T}{4\;N²}$$

where L is the radiance map from the scene (W/(m^2^ sr)), T is the transmission factor of the objective and the optical filter (between 0 and 1), and N is the aperture (f-number). The transmission factor depends on the wavelength. 

Equations (2) and (3) demonstrate a proportional relation between the exposure and the radiance maps, given a fixed configuration of the camera. Consequently, it is possible to build a response curve integrating not only the effects of camera electronics but also the effects of the optics. For this, we define the exposure map at the entrance of the objective:

(4) $$H_{\mathrm{lens}}=L\;t$$

**Figure 1:**
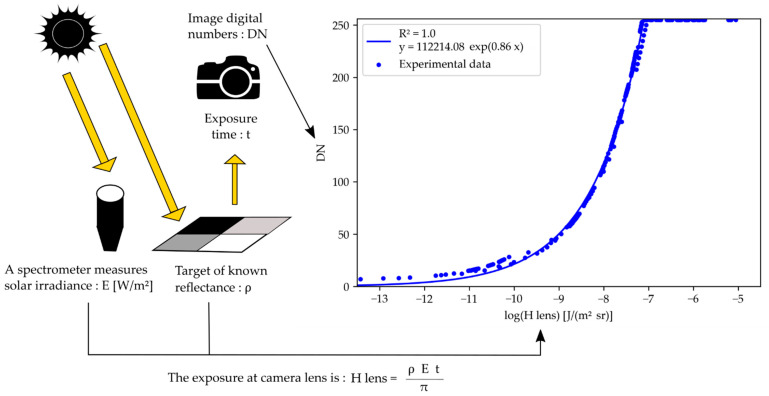
Method to build a camera response curve linking the image digital numbers and the exposure at camera lens. The curve used as example is the response curve of the 490 nm camera.

The modified response curve expresses the relation between the DN and that exposure at the entrance of the objective. To build that curve, it is necessary to obtain $H_{\mathrm{lens}}$ values for a range of DN values. For this, Equations (1) and (4) are combined:

(5) $$H_{\mathrm{lens}}=\frac{\rho \;E\;t}{\pi }$$

where ρ is the known reflectance of a reference panel, E is measured by the ILS, and t is recorded at image acquisition. The method to build the modified camera response curve is represented in Figure 1. For each camera of the multispectral array, fifty images of the panel were acquired under natural light. A different exposure time value was set for each image. That way, while exploiting the four reflectance targets, it was possible to generate a wide range of DN values. The position of the four targets was automatically extracted in each image thanks to an ArUco marker in the panel suitcase. A spectrum of the incident light was recorded at the time of each image acquisition. That spectrum was integrated over the wavelength bands corresponding to the camera filters, considering the transmission of an ideal filter. Similarly, the reference reflectance spectrum of the panel was integrated in the wavelength bands of the filters. Using those values and equation 5, it was possible to compute the $H_{\mathrm{lens}}$. Then, the $H_{\mathrm{lens}}$ values and the DNs extracted from the images were used to build the response curve. The DN-intercept was imposed at 0 for fitting the model. This choice was made to avoid posterior issues when exploiting the curve in case DN values were smaller than a non-zero DN-intercept. 

#### 2.3.3. Wheat Organ Bi-Directional Reflectance Factor (BRF)

**Figure 2:**
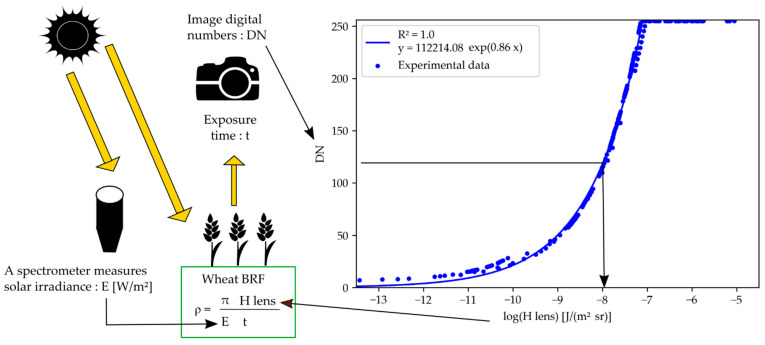
Method to compute wheat bi-directional reflectance factor (BRF) from image digital numbers and ILS readings. The method exploits the camera response curve. $H_{\mathrm{lens}}$ is the exposure at camera lens.

For each channel, average DN values were computed for the segmented wheat organs. Those DNs were converted to exposure values through the camera response curves and the recorded exposure times. The sunlight irradiance spectra measured by the ILS were integrated over the wavelength bands of the cameras filters and combined with exposure values to compute the reflectance of the plant organs. The method is represented in Figure 2. 

### 2.4. Images Registration

The multispectral camera array images and the RGB images were registered using the B-SPLINE method described by [54]. After this operation, the images could be aligned pixel-to-pixel to form a single multi-channel image. A 5 × 5 blur was applied on each channel to diminish the impact of potential registration errors.

### 2.5. Image Segmentation at the Organ Scale

Three classes were considered for segmentation: the background (the soil), the leaves plus the few stems visible in the images, and the ears. The segmentation process was performed in one or two steps, depending on whether the image contained ears. The first step was the segmentation between the background and all the wheat organs. The separation used a threshold in the 800 nm channel, which was automatically determined for each image based on the first local minimum of the histogram. That simple method worked because of the significant reflectance difference of the wheat and the soil in the near-infrared. However, in some cases where direct sunlight reached the soil, a few soil pixels could be confused with the low and shaded leaves. To avoid this error, two additional thresholds were applied for the pixels of low values in the 800-nm channel: a threshold in the blue channel of the multispectral camera array and a threshold on the Excess Red index [55] computed from the green and red channels of one RGB camera. The need to add these thresholds was determined thanks to a cloudiness index derived from the ILS data at the time of image acquisition. The index was formulated as:

(6) $$C_{T}=1-\frac{E}{E_{0}\;\cos\left(z\right)}$$

where E is the solar irradiance (W/m²) in the spectral measurement range of the ILS, $E_{0}$ is the solar constant (1360 W/m²), and z is the sun zenith angle. An empirically found threshold of 0.90 was exploited to determine the conditions of strong direct sunlight.

**Figure 3:**
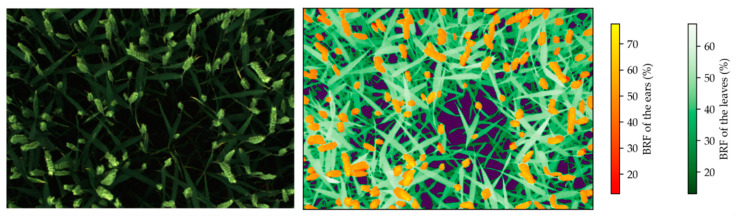
To the left, a registered RGB image. To the right, the corresponding registered and segmented bi-directional reflectance factor (BRF) map in the 800 nm channel. The color bars indicate the BRF values separately for ears and leaves. The dark purple regions in the image represent the background.

The second segmentation step, when ears were in the imaged scene, was used to separate the ears and the rest of the image. That segmentation was performed using the deep learning approach detailed by [56]. First, the YOLOv5 model (DOI: 10.5281/zenodo.3908559, accessed on 13 November 2021) detected the ear bounding boxes. Second, the DeepMAC model [57] segmented the ears in the bounding boxes. Once the ears, leaves, and background were separated, their masks were applied on the BRF maps to extract BRF values for the different wheat organs. An example of segmented BRF map is presented in Figure 3.

## 3. Results and Discussion

### 3.1. Camera Response Curves

**Figure 4:**
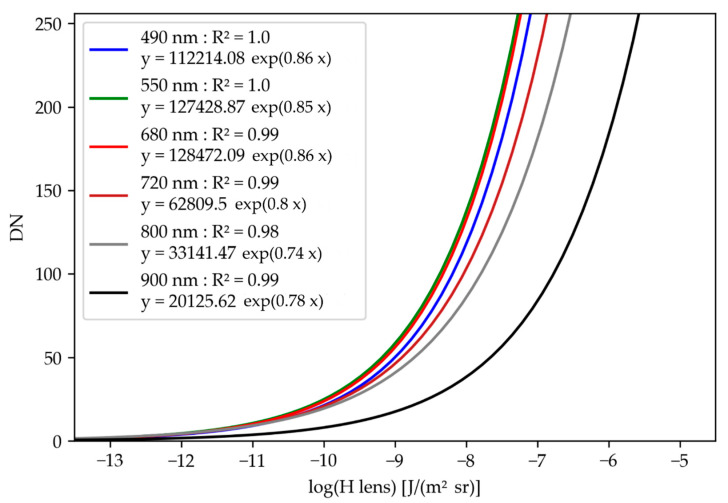
Response curves of the monochrome cameras. Each camera is designated using the central wavelength of the narrow-band optical filter. The response curves model the relation between image digital numbers (DNs) and exposure at camera lens ($H_{\mathrm{lens}}$).

The response curves of all the monochrome cameras are presented in Figure 4. The determination coefficients demonstrate that the curves were well-adjusted on the measurement points for all the cameras. The differences between the curves can be explained by the relative sensitivity of the sensors to the wavelength and the different transmissivities of the optical filters. 

### 3.2. Bi-Directional Reflectance Factor of the Reference Panel

**Figure 5:**
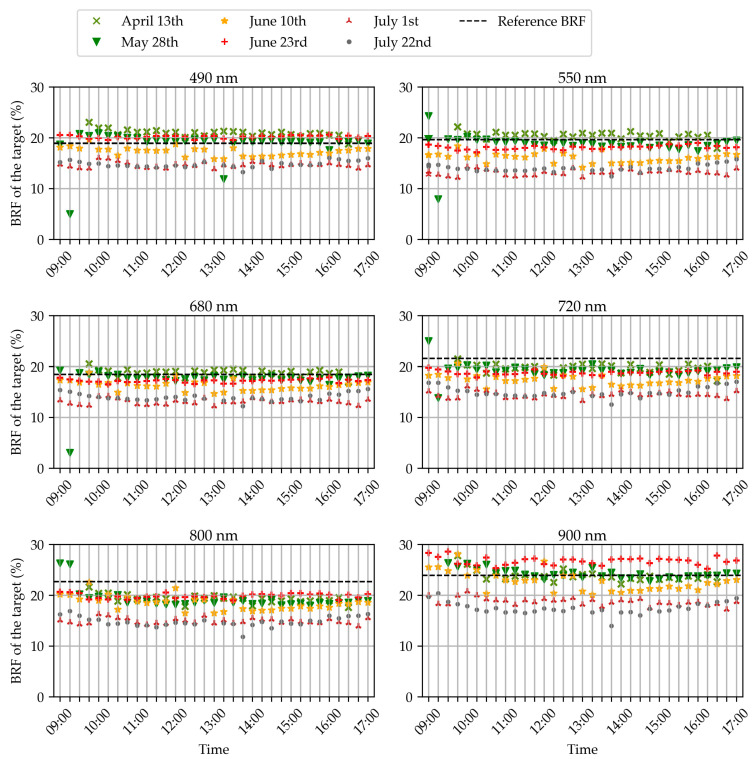
Evolution throughout the day of the average bi-directional reflectance factor (BRF) measured on the dark gray target. Each subplot is dedicated to a camera of the multispectral array, designated by the central wavelength of its optical filter. The evolution of the measured BRF is represented for six different acquisition dates, indicated by a color and symbol code on each subplot. The dotted line corresponds to the theoretical BRF of the target that was provided by the manufacturer.

Figure 5 presents the evolution of the BRF measured throughout the day on the dark gray reference target. The evolution was studied for the six filters and the six all-day-long acquisition dates. 

A first important observation is that, in all the scenarios, the measured BRF was roughly constant throughout the day. This proves that the method based on the spectrometer was able to compensate for the relative changes in scene illumination despite major variations on the concerned days. The few outliers (28 May at 9 and 9.15 a.m. in Figure 5) can be attributed to errors in the automatic detection of the reference panel. The reflectance was influenced by some pixels not belonging to a target of the panel but considered as such in the segmentation process. 

A second important observation is that the measured BRF was not always equal to the reference value provided by the manufacturer. This highlights that the method cannot reliably retrieve accurate absolute BRF values. The differences from the reference are clearly related to the acquisition date, and sensor drift is unlikely because the importance of the variations is not related to a chronological order. The first obvious explanation could be the illumination conditions. In the literature, ref. [58] measured the reflectance spectra of reference targets and pointed out differences between three days of different weather conditions. For our study, the response curves of the cameras were established under very diffuse light conditions. This could explain an underestimation of the BRF on sunny days such as 10 June and 22 July. Nevertheless, variations of sky conditions were also observed within the same date, e.g., on 1 July when the light was almost totally diffuse in the morning but the sky cleared up in the afternoon, and no major variations of BRF occurred within those dates. For this reason, our hypothesis is that the differences between dates were generated by other factors than the illumination conditions. We suspect that the acquisition system configuration has a role. Potential sources of variations were the orientation of the cosine corrector of the spectrometer; the connection between the optical fiber, the cosine corrector, and the spectrometer; the orientation of the cameras; the orientation of the target; and the distance between the cameras and the target. 

Similar observations were made for the light gray and black targets, except that for the black target the differences were mainly due to slight overestimations. The white target could not be used because it appeared saturated in some images. This saturation was due to the auto-exposure algorithm of the Micro-MCA camera array. The exposures from the five slave cameras were computed according to the auto-exposure of the 800 nm camera, referred as the master. The relations between the master and the slaves, called relative exposures, were tuned to suit the light reflected from the wheat in the different wavelengths, not from the target. 

### 3.3. Wheat Organ Bi-Directional Reflectance Factor (BRF) throughout the Day

**Figure 6:**
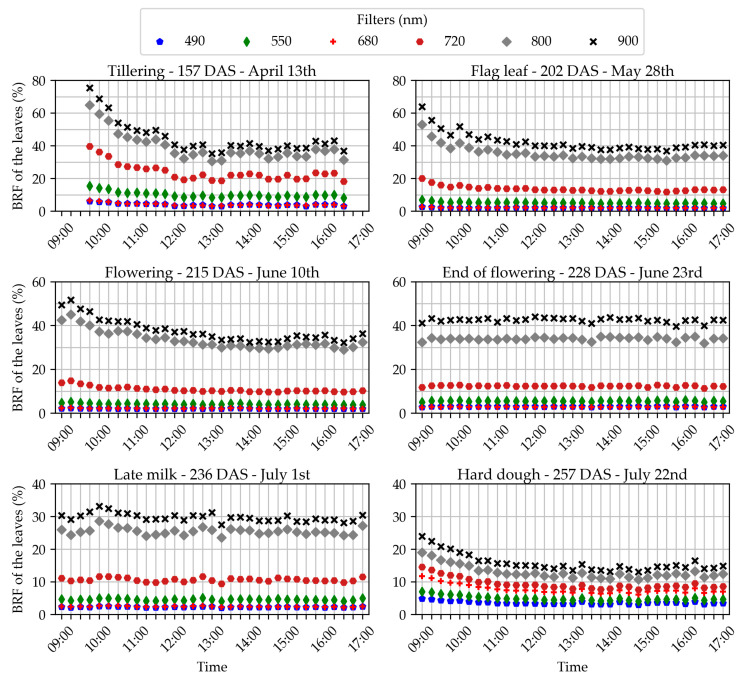
Evolution throughout the day of the average bi-directional reflectance factor (BRF) measured on the leaves. Each subplot is dedicated to an acquisition date, designated by the development stage of the crop and the number of days after sowing (DAS). The evolution of the measured BRF is represented for the six different optical filters of the cameras, indicated by a color and symbol code on each subplot.

Figure 6 presents the evolution of the BRF measured throughout the day on wheat leaves. The evolution was studied for the six filters and the six all-day-long acquisition dates. The goal was to study the robustness of leaf BRF measurements. It was assumed that no change of BRF would occur during the day because of plant physiology or canopy architecture variations. For most dates, the BRF gradually decreased in the morning and then was roughly constant in the afternoon. We hypothesize that the sun zenith angle had an influence. Indeed, the effect was the strongest on 13 April, and at this period of the year, the sun was really low on the horizon in the morning. On the contrary, no change of BRF was observed on 23 June, which was the moment in the year where the sun was the highest in the morning and specifically very cloudy day. Similarly, 1 July in the morning was also cloudy, which may explain why the sun zenith angle had no effect. On the concerned dates, solar noon varied from 13:38 to 13:47 h. The best acquisition time to obtain comparable BRF measurements seems to be in the hours close to solar noon. Moreover, these acquisition hours minimize the impact of shadows. Rather than only focusing on hours, the sun zenith angle should be examined. Periods when the days are longer and the sun is higher would permit a wider range of acquisition hours. The data suggest that a zenith angle above 55° could secure comparable wheat BRF measurements for a camera in the nadir position. It also seems that the azimuth had no impact on the BRF of wheat leaves, which was expected because (i) the crop was dense with barely visible rows and (ii) the cameras captured the crop from nadir. For the hours when the sun was high enough, the BRF showed almost no variations, which proves the strong quality of the method and confirms the hypothesis that no variations due to physiology or canopy architecture occurred during these hours. For 10 June, 23 June, 1 July, and 22 July, the same study was carried out on the ears in addition to the leaves. The curves showed the same trends except on 10 June, when the ear BRF curve was bowl-shaped, with a minimum around solar noon. 

### 3.4. Wheat Organ Bi-Directional Reflectance Factor (BRF) in Fertilization Trials

The results from Section 3.2 and Section 3.3 indicate that the computed wheat BRF values were comparable within the same date if the sun was high enough or if the sky was cloudy. However, different dates presented slight variations in absolute BRF values, probably because of the configuration of the acquisition system. To deal with this effect, the values of the dark gray target on the reference panel were used to correct the wheat organ average BRF. Each wheat BRF value was multiplied by the ratio of the BRF measured on the target at this date to the theoretical reference BRF of the target. In this multiplication factor, the BRF measured on the target was the mean of the values measured at the beginning and the end of the acquisition of all wheat micro-plots. In the rare cases where the difference between the BRF of the target at the beginning and at the end was larger than 3%, only the value closest to the theoretical BRF was considered. The furthest value was considered to be unreliable. The difference of reference BRF values within a same date were probably due to the positioning of the panel in the field. Less care was used during this step than in the all-day-long experiments. Here, the reference panel was held by human hands or dropped above the canopy. The two parts of the open suitcase containing the panel and the ArUco marker for panel segmentation could have been folded, modifying the relative position of the panel and the marker, thus the leading to a panel segmentation error. A wheat leaf could also have been moved above the panel at the time of acquisition. 

**Figure 7:**
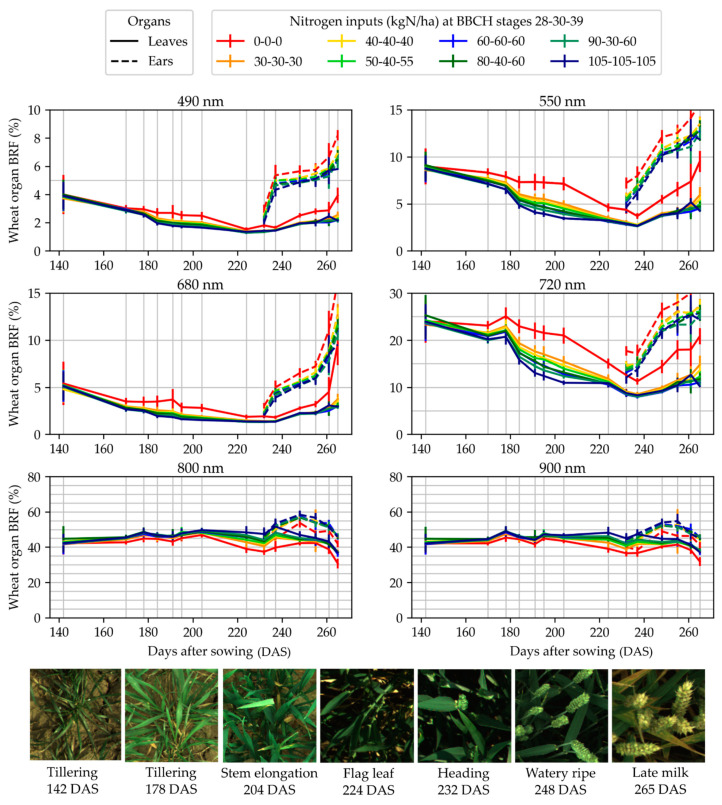
Temporal evolution of the measured bi-directional reflectance factor (BRF) during the measurement campaign in trial 1. The evolution is presented for the six monochrome cameras, designated by the central wavelength of their narrow-band optical filter.

**Figure 8:**
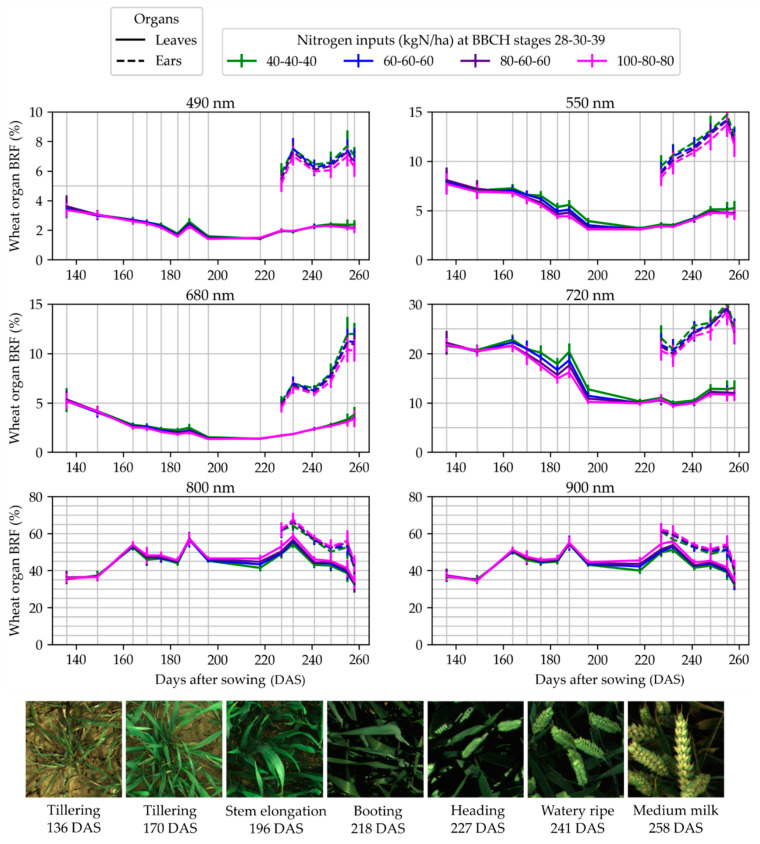
Temporal evolution of the measured bi-directional reflectance factor (BRF) during the measurement campaign in trial 2. The evolution is presented for the six monochrome cameras, designated by the central wavelength of their narrow-band optical filter.

Figure 7 and Figure 8 present the dynamics of the average BRF measured in the fertilization trials and corrected using the BRF of the reference panel measured at the beginning and the end of the acquisition. For both trials, the curves show a coherent evolution, i.e., the points could be distributed on a smooth line. The same trends were observed for all the fertilization objects and the BRF values stratified along with the fertilization level. Those elements validate the consistency of the method. In Figure 7, a suspected increase in BRF can be observed for the 105-105-105 kgN/ha fertilization object at 261 days after sowing (DAS) for the visible and red edge wavebands. This spike is probably due to the mass lodging on the micro-plots of that fertilization object. Almost all the plants were lying on the ground, so the images had to be taken in the borders of the micro-plots, where their nutrition and development could have been different. Some plants were also tilted in those images. 

## 4. Conclusions

This paper detailed an automatic method to compute the bi-directional reflectance factor (BRF) of wheat while disentangling the contribution of leaves and ears. The method was found to allow for high-throughput measurements and did not necessitate the positioning of a reference panel in all imaged scenes. Multispectral images were acquired at the same time as incident light spectra. First, image digital numbers were converted to BRF values by exploiting camera response curves and the incident light integrated in the wavelength bands corresponding to the camera filters. Second, the BRF maps were segmented to separate the background, the leaves, and the ears. 

The BRF measured on reference targets was robust throughout the day but showed variations with the acquisition date. As a consequence, when measuring the BRF of wheat micro-plots at several dates, the reference panel needs to be imaged at least once during each date. In this study, the panel was captured at the beginning and the end of the acquisitions, and it was used to correct the absolute BRF values. All over the wheat growing season, the method yielded consistent BRF values that were in good agreement with the fertilization levels. Regarding the BRF of leaves and ears throughout a day, constant values were recorded in cloudy conditions or close to solar noon. However, the organ BRF gradually changed in the morning under sunny or partially cloudy skies. This suggests that images for robust BRF measurement should preferably be acquired under cloudy conditions or close to solar noon. 

In a broad context, this method will ease the high-throughput measurement of spectral features of wheat corrected for variations in the sunlight spectrum and promote their extraction at the organ scale in field conditions.

## Figures and Tables

**Table 1:** Dates of all-day-long acquisition.

Date	Days after Sowing	BBCH Growth Stages	Sky Conditions	
			Morning	Afternoon
13 April	157	29: Tillering	Sunny	Cloudy
28 May	202	39: Flag leaf	Sunny	Sunny
10 June	215	65: Flowering	Sunny	Sunny
23 June	228	69: End of flowering	Heavy clouds	Heavy clouds
1 July	236	77: Late milk	Heavy clouds	Sunny
22 July	257	87: Hard dough	Sunny	Sunny

## Data Availability

The data presented in this study are available on request from the corresponding author.

## Appendix A

To prevent saturation, a custom auto-exposure algorithm was designed for the RGB cameras. A dichotomous search was performed to find the highest exposure time for which no saturation was detected in the image. The two limits of the search were the exposure time computed by the manufacturer algorithm and this time divided by five.

For the multispectral camera array, the factory auto-exposure algorithm computed the exposure time for a master camera and multiplied this time by pre-defined coefficients, called relative exposures by the camera manufacturer, to obtain the exposure times for the other cameras. The images from the master camera were never over-exposed, but the images from the slave cameras could have been too bright at some acquisition dates if the multiplication coefficients were not tuned. Indeed, the proportion of light reflected from the scene in the different wavebands of the multispectral device was affected by the development stage of the crop. To ensure proper exposure, the coefficients determining the exposure times of the slave cameras were adapted three times during the season. The first set of coefficients suited most of the growing season, when most of the pixels corresponded to green plant elements, but a second, third, and fourth set were necessary to account for the maturity of the ears (Table A1). The coefficients were empirically determined.

**Table A1:** Suitable relative exposures for the six wavebands of the Micro-MCA multispectral camera array depending on the BBCH development stage. Those values prevented saturation in the acquired images.

BBCH	Relative Exposures in the Six Wavebands of the Multispectral Array					
	490 nm	550 nm	680 nm	720 nm	800 nm	900 nm
32 to 75	400	250	400	200	80	220
77	250	100	250	150	80	200
83 to 87	160	100	100	100	80	140
89	110	70	60	100	80	140
